# Intrathyroidal Thymoma: A Diagnostic Challenge

**DOI:** 10.7759/cureus.68987

**Published:** 2024-09-09

**Authors:** Adhithya N Balaji, Balaji Balasubramanian, Juman A Hazeem

**Affiliations:** 1 Oncology, Università Cattolica del Sacro Cuore, Rome, ITA; 2 Surgery and Oncology, NMC Specialty Hospital, Abu Dhabi, ARE; 3 Pathology, NMC Royal Hospital, Abu Dhabi, ARE

**Keywords:** aberrant thymic tissue, ectopic thymoma, fnac, immunohistochemistry, intrathyroidal thymoma, thymoma, thyroid

## Abstract

Intrathyroidal thymoma is a rare tumor that can be challenging to diagnose due to its unusual location and resemblance to more common thyroid conditions. We present the case of a 58-year-old woman with an incidentally discovered thyroid nodule during evaluation for an upper respiratory infection. Ultrasonography revealed an exophytic nodule in the left thyroid lobe, categorized as TR 3. Fine-needle aspiration cytology suggested a neoplastic process, leading to a left hemithyroidectomy. Histopathology confirmed a diagnosis of intrathyroidal thymoma, Type B2, with extensive necrosis, and immunohistochemistry validated the findings. This case underscores the diagnostic challenges of intrathyroidal thymoma, emphasizing its consideration in the differential diagnosis of atypical thyroid nodules. Despite the difficulties in preoperative identification, surgical resection and subsequent histopathological examination remain essential for a definitive diagnosis. The patient is currently under surveillance, and there is no evidence of residual thymic tissue or abnormalities in the remaining thyroid tissue.

## Introduction

Intrathyroidal thymoma is a rare clinical entity usually diagnosed postoperatively from the histopathological studies of the excised specimen. In this case report, we describe a 68-year-old Filipino woman who presented to us with a thyroid nodule. After the final histopathological study, an ectopic intrathyroidal thymoma was diagnosed. The case is discussed further due to the rarity of the presentation.

Thymoma can arise in the thyroid gland, possibly due to the aberrant thymic tissue left in the thyroid gland [1]. It can mimic a carcinoma, which has a poorer prognosis. Thymomas are epithelial tumors arising from the thymus gland, normally in the anterior superior mediastinum. Rarely, it can occur at an ectopic location from an aberrant thyroid tissue due to displaced thymic tissue during embryological development. The incidence of ectopic thymomas is quite rare, ranging up to 4% of all thymomas [2]. The site includes the neck, mediastinum, lung, and pleural cavity. Primary intrathyroidal thymoma is differentiated from ectopic thymoma due to the presence of thymic content inside the thyroid gland. In the past, primary intrathyroidal thymoma included a group of neoplasms, SETTLE (spindle epithelial tumor with thymus-like differentiation) and CASTLE (carcinoma showing thymus-like differentiation) [3].

## Case presentation

A 58-year-old Filipino woman was incidentally found to have a thyroid nodule while she was being treated for an upper respiratory tract infection.

Radiological findings

**Figure 1:**
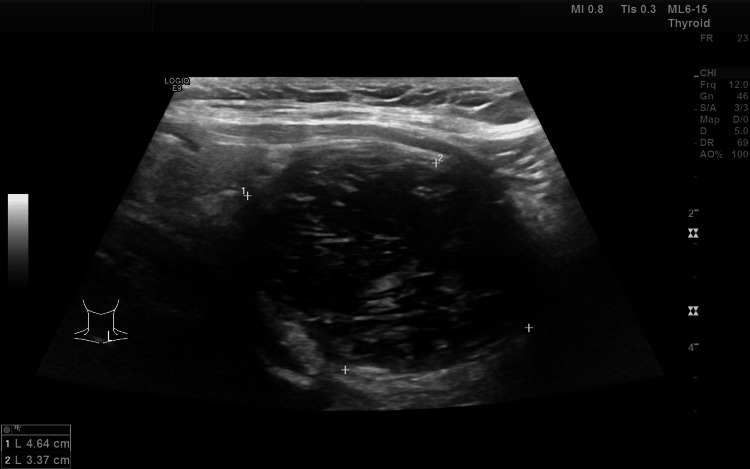
Ultrasound (US) image of the thyroid gland showing an exophytic nodule measuring 4.6 x 3.4 cm, having a solid and cystic component with a spongiform appearance arising from the lower pole of the left lobe of the thyroid gland.

An ultrasound (US) of the thyroid gland was performed, which revealed an exophytic nodule measuring 4.6 cm × 3.4 cm, arising from the lower pole of the left thyroid lobe, classified as a TR 3 nodule (Figure 1). A tiny cyst measuring 1.7 mm was noted in the midpole of the right lobe of the thyroid gland, classified as a TR 1 category. In addition, a spongiform nodule measuring 0.6 cm × 0.4 cm was noted in the lower pole of the left thyroid lobe, classified as a TR 2 category. X-rays of the chest and neck were done, which were within normal limits.

Lab and cytological findings

**Figure 2:**
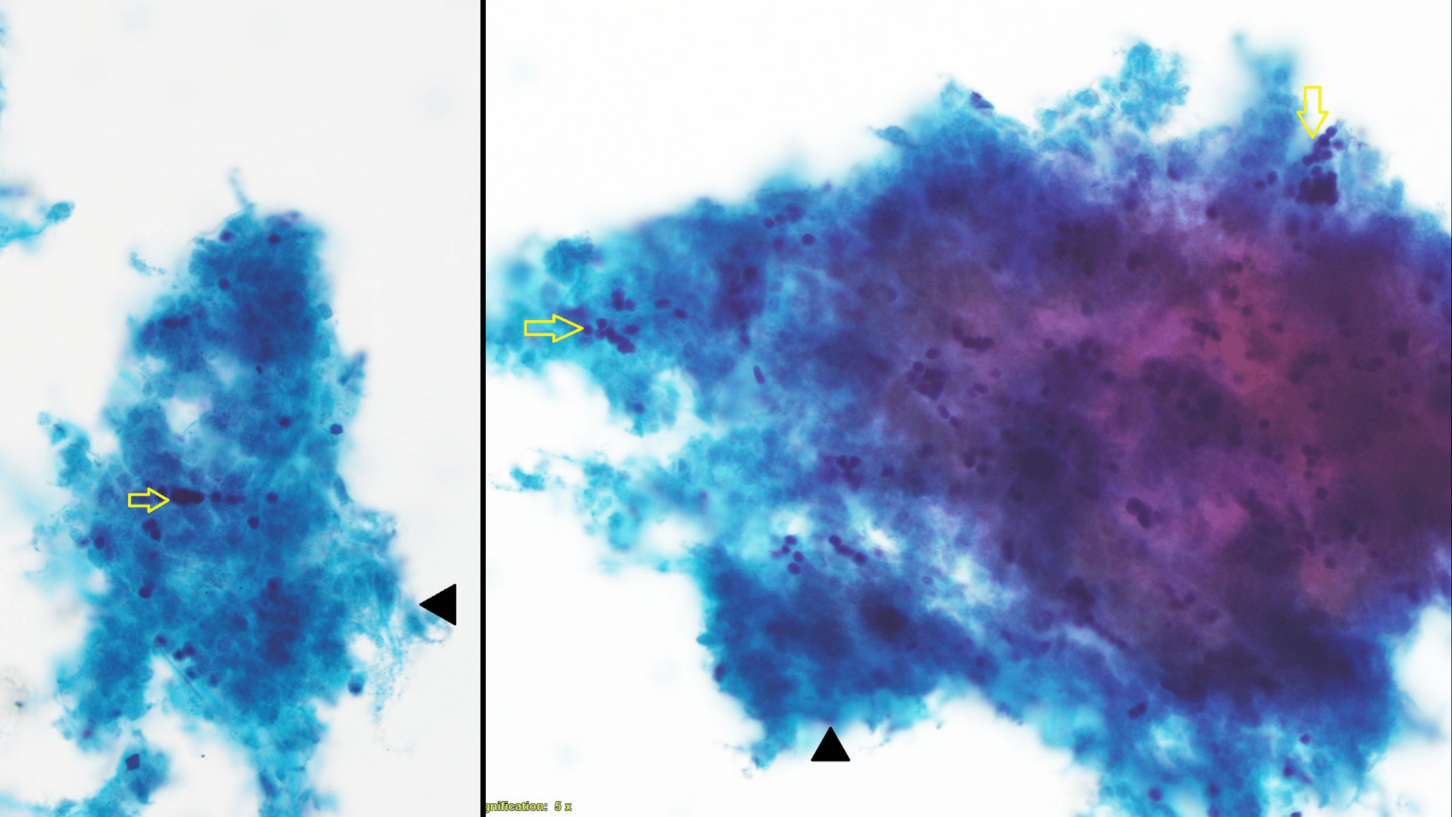
FNAC of the left thyroid nodule reveals areas of lesional cell necrosis (black arrowhead) adhering to small hyperchromatic nuclei (yellow arrow) without clear cytoplasm. Magnification: 400× FNAC: fine-needle aspiration cytology

The patient's thyroid function was found to be normal. US-guided fine-needle aspiration cytology (FNAC) was done from the TR 3 nodule. FNAC showed necrosis with a few small hyperchromatic nuclei, showing no follicular cells (Figure 2). With a diagnosis of a solitary thyroid nodule arising from the lower pole of the left lobe of the thyroid gland, it was decided to go ahead with surgery to rule out follicular neoplasm or poorly differentiated thyroid carcinoma. The patient underwent a left hemithyroidectomy.

Surgical findings

**Figure 3:**
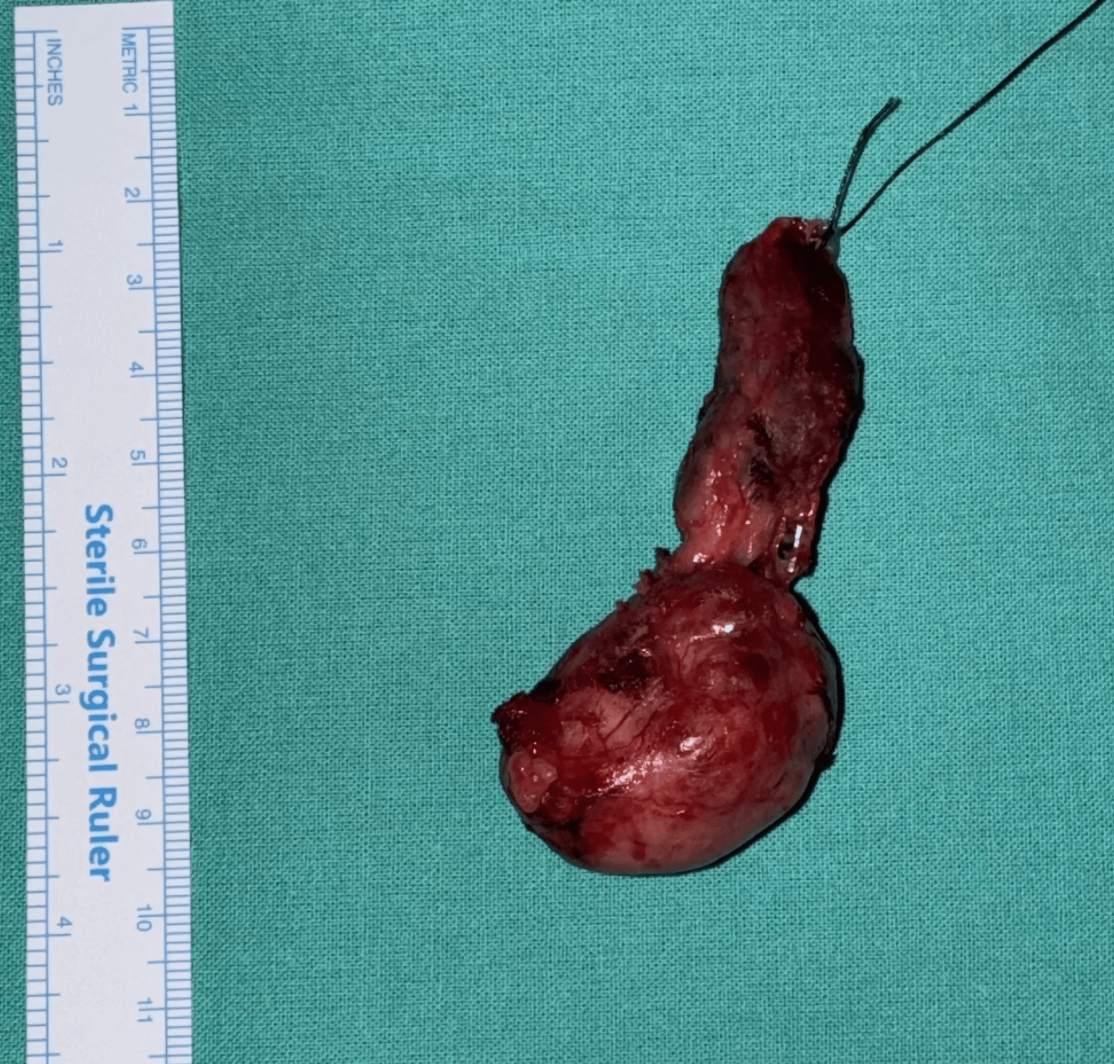
The surgical specimen from the left hemithyroidectomy, measuring 7 × 2.5 × 2 cm and weighing 16 g.

During surgery, the nodule seemed to arise from the lower pole of the left lobe of the thyroid gland, and it exhibited retrosternal extension for approximately 2.5-3 cm, which was excised completely (Figure 3). No extrathyroidal extension was observed during surgery. There were no suspicious lymph nodes. The recurrent laryngeal nerve was identified and safeguarded. The right lobe was found to be normal.

Histopathological findings

Gross Findings

The left hemithyroidectomy specimen weighs 16 g and measures 7 x 2.5 x 2 cm. The cut section shows a nodule measuring 4 x 2.5 x 2 cm in the inferior aspect of the gland.

Microscopical Findings

**Figure 4:**
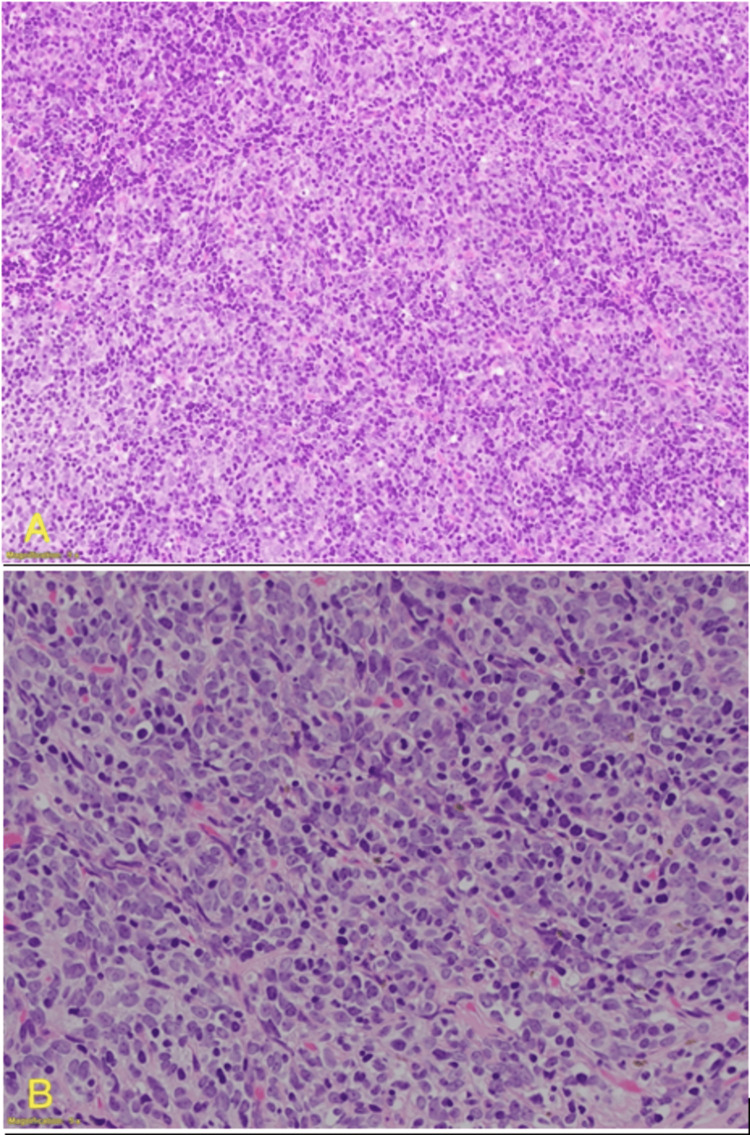
Histopathology of the nodule stained with H&E showing no follicular cells. (A) 200× magnification; (B) 400× magnification H&E: hematoxylin and eosin

This is an intrathyroidal thymic thymoma Type B2 (no medullary islands) with 90% extensive coagulative tumor necrosis (Figure 4) and a focal margin status of less than 0.5 mm, pT1aNxMx.

Immunohistochemistry

**Figure 5:**
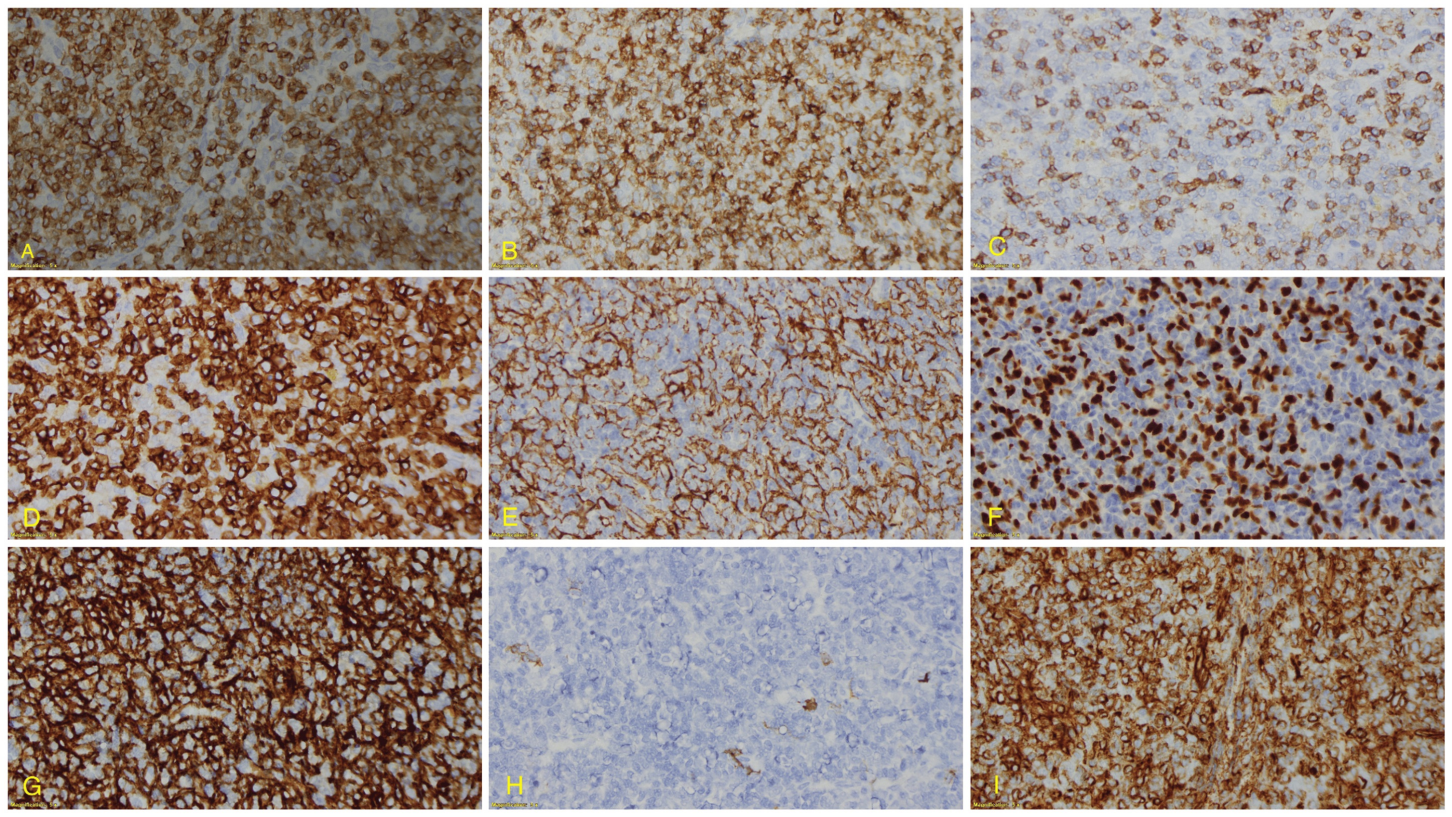
Immunohistochemistry panel of the nodule with the following stains: (A) CD3, (B) CD5, (C) CD45, (D) CD99, (E) CK 5/6, (F) p63, (G) CK (AE1/AE3), (H) CD20, and (I) vimentin. Immunohistochemistry of the nodule showed positivity with the following stains: (A) CD3 (polyclonal), (B) CD5 (4C7), (D) CD99 (12E7), (E) CK 5/6 (D5/16 B4), (F) p63 (Dak-p63), (G) CK (AE1/AE3), and (H) CD20 (L26), while it showed patchy positivity with (I) vimentin (V9)

The immunohistochemistry analysis of the ectopic intrathyroidal thymoma revealed the following findings: cytokeratin (AE1/AE3), showing a diffuse meshwork of neoplastic epithelial cells, was positive both in viable thymoma cells and areas of extensive necrosis (Figure 5G). CK 5/6 (D5/16 B4) and p63 (Dak-p63), which are markers for squamous differentiation and vimentin (V9), showed positivity in viable thymoma cells and necrotic regions, indicating epithelial and mesenchymal differentiation (Figures 5E, 5F, 5I). CD117 (EP10) and TTF-1 (SPT24) were negative, helping to rule out differential diagnoses such as gastrointestinal stromal tumors and thyroid neoplasms. CD5 (4C7) positivity was noted in viable thymoma cells (Figure 5B), further supporting the diagnosis of thymoma. CD3 (polyclonal) (Figure 5A) and CD20cy (L26) demonstrated positive staining in viable thymoma and focal areas of necrosis (Figure 5H), while BCL2 oncoprotein (124) showed positivity in scattered cells within the tumor. CD45, LCA (2B11+PD7/26) showed patchy positivity (Figure 5C), and CD99 (12E7) was positive in viable thymoma with focal staining in necrotic regions (Figure 5D). Ki-67 (MIB-1) was markedly increased in viable thymoma cells, indicating high proliferative activity. These findings collectively support the diagnosis of an ectopic intrathyroidal thymoma with areas of extensive necrosis.

Further plan

The case was further discussed in the multidisciplinary tumor board. Computed tomography (CT) scans of the neck and chest were performed. No residual thymic tissue was found in the mediastinum, and the right thyroid lobe was found to be normal. Therefore, it was decided to keep her under follow-up.

## Discussion

The pyramidal-shaped thymus gland consists of two incompletely fused lobes with a fibrous capsule with an apex extending to the inferior pole of the thyroid and the base extending to the great vessels in the mediastinum. Histologically, it contains primarily T lymphocytes and epithelial cells.

Embryologically, the thymus is derived from the third and fourth pharyngeal pouches. During the initial weeks of gestation, the primitive thymic tissue descends from the pharynx to the anterior mediastinum along the thymopharyngeal tract [4]. It is similar to the thyroglossal duct for the thyroid gland. It eventually settles down in the anterior mediastinum, while the thyroid gland settles in the lower neck. Either inappropriate descent or sequestration or persistent thymic nest of cells in this tract can lead to an ectopic thymoma in the future. This has been identified in a very small group of patients. The occurrence of primary intrathyroidal thymoma is a highly uncommon phenomenon, and only five such cases have been reported in the literature [2].

Thymomas are tumors arising from the thymic epithelial origin, which have a mixture of neoplastic epithelial cells and non-neoplastic immature T lymphocytes [5]. They may be fully encapsulated or of an invasive type. Encapsulated thymomas are more common, primarily seen in the anterior mediastinum. Rarely, an ectopic thymoma can be seen in the neck, submandibular region, cervical region, pleura, or in the pulmonary hilum up to the inferior mediastinum close to the diaphragm [6]. In such cases, these thymomas are likely to arise from the vestigial remnants along the thymopharyngeal duct [5].

Historically, Bowman described the case of ectopic thymoma in the cervical region in 1941 [5]. This rare tumor appears seven times more commonly in females (7:1 ratio). In the neck, ectopic thymoma is found in the lower neck anterolaterally, adjacent to the lower pole of the thyroid gland or within the lower pole of the thyroid tissue itself, as in our case [3]. Primary intrathyroidal thymoma is usually not associated with myasthenia gravis or any other autoimmune disorder [2].

The usual differential diagnosis includes Hashimoto's thyroiditis or lymphoma in the thyroid gland [7]. Interpretation of the FNAC can be misleading, and it rarely has to be differentiated between carcinomas showing thymus-like differentiation. Most of these reported cases suggest a benign course, which is cured with complete surgical resection [5]. It gives a diagnostic challenge while interpreting FNAC. The first case series with three cases of intrathyroidal thymoma was published in 1985 [1].

The diagnosis of intrathyroidal thymoma can be very challenging, and most of the cases are diagnosed after surgical resection. Imaging shows only a thyroid nodule; no specific characteristic feature is assigned to a thymoma in the thyroid gland. Interpreting the FNAC can be challenging, leading to a diagnostic difficulty. A differential diagnosis of Hashimoto's thyroiditis or a low-grade lymphoma may be considered due to the atypical lymphoid proliferation. The ultimate diagnosis can be made only after a surgical resection. Even the frozen section has been very misleading in the few reported cases [8].

Final histopathology might be challenging as intrathyroidal thymomas can mimic an anaplastic carcinoma or a lymphoma. Lymphoepithelioma-like thymic carcinoma can morphologically resemble an intrathyroidal thymoma, and it should be differentiated by its high-grade cytological features with squamoid differentiation and mature lymphocytic element. The other challenge in differential diagnosis could be an insular carcinoma of the thyroid [9]. Primary intrathyroidal thymoma looks similar to its mediastinal counterpart in terms of both morphology and immunohistochemistry. Complete surgical resection appears to be the treatment of choice as long as the lesion is usually confined to the thyroid gland [2].

In summary, an appropriate histopathological diagnosis and complete excision are the keys to the management of primary intrathyroidal thymoma. It is a rare entity, and clinicians need to be aware of this condition.

## Conclusions

Intrathyroidal thymoma represents a rare clinical possibility of an ectopic thymoma. The challenge lies in the diagnosis of the entity. The FNAC can be inconclusive and suggestive of a differential diagnosis between Hashimoto's thyroiditis and lymphoma. Surgical removal and a complete histopathological examination will give the final diagnosis, and awareness about this rare entity will raise clinical suspicion while evaluating such rare conditions.
